# Yeast Particle Encapsulation of Scaffolded Terpene Compounds for Controlled Terpene Release

**DOI:** 10.3390/foods10061207

**Published:** 2021-05-27

**Authors:** Ernesto R. Soto, Florentina Rus, Hanchen Li, Carli Garceau, Jeffrey Chicca, Mostafa Elfawal, David Gazzola, Martin K. Nielsen, Joseph F. Urban, Raffi V. Aroian, Gary R. Ostroff

**Affiliations:** 1 Program in Molecular Medicine, University of Massachusetts Medical School, Worcester, MA 01605, USA; ernesto.soto-villatoro@umassmed.edu (E.R.S.); florentina.rus@umassmed.edu (F.R.); hanchen.li@umassmed.edu (H.L.); carli.garceau@umassmed.edu (C.G.); jeffrey.chicca@umassmed.edu (J.C.); mostafa.elfawal@umassmed.edu (M.E.); davidmgazzola@gmail.com (D.G.); raffi.aroian@umassmed.edu (R.V.A.); 2 M.H. Gluck Equine Research Center, Department of Veterinary Science, University of Kentucky, Lexington, KY 40546, USA; martin.nielsen@uky.edu; 3 Beltsville Human Nutrition Research Center, Diet, Genomics and Immunology Laboratory, United States Department of Agriculture, Agricultural Research Service, Beltsville, MD 20705, USA; joe.urban@usda.gov; 4 Beltsville Agricultural Research Center, Animal and Parasitic Diseases Laboratory, United States Department of Agriculture, Agricultural Research Service, Beltsville, MD 20705, USA

**Keywords:** terpenes, yeast particles, antimicrobial, anthelmintic, prodrug

## Abstract

Terpenes are naturally occurring compounds produced by plants that are of great commercial interest in the food, agricultural, cosmetic, and pharmaceutical industries due to their broad spectra of antibacterial, antifungal, anthelmintic, membrane permeation enhancement, and antioxidant biological activities. Applications of terpenes are often limited by their volatility and the need for surfactants or alcohols to produce stable, soluble (non-precipitated) products. Yeast particles (YPs) are hollow, porous microspheres that have been used for the encapsulation of terpenes (YP terpenes) by passive diffusion of terpenes through the porous YP cell walls. We here report the development of a second generation YP encapsulated terpene technology that incorporates the stimuli-responsive control of terpene release using biodegradable pro-terpene compounds (YP pro-terpenes). YP terpenes and YP pro-terpenes were both produced, in which high levels of carvacrol, eugenol, thymol and geraniol were encapsulated. The YP pro-terpenes show higher encapsulation stability than YP terpenes due to pro-terpenes being non-volatile solids at room temperature and stable in suspensions at neutral pH. YP pro-terpenes and YP terpenes were evaluated for biological activity in antibacterial, antifungal and anthelmintic assays. The YP pro-terpenes retained the full biological activity of the parent terpene compound.

## 1. Introduction

Terpenes are a large class of naturally occurring organic compounds that constitute primary components of essential oils obtained from plants. The term terpene is commonly used to include basic terpenes or hydrocarbons consisting of isoprene repeating units and terpenoids or modified terpenes containing additional functional groups (usually oxygen-containing groups). Terpenes have long been recognized for a wide range of functional properties (e.g., anthelmintic, antifungal, antibacterial, and antioxidant properties). Terpenes are used as excipients due to their permeation enhancer properties, or as bioactive compounds in pharmaceutical products, as fragrances, permeation enhancers and antioxidants in cosmetics, for their broad range of potential applications against pathogens in agricultural products, and can be used as additives in food packaging to prevent bacterial spoilage and oxidation [1,2,3].

The use of terpenes can present challenges primarily associated with (1) the chemical instability of some isolated terpenes when exposed to air, heat, light and moisture; (2) poor water solubility, and (3) high volatility. It is usually necessary to produce terpene-based products containing high levels of surfactants or alcohols. Applications of terpenes in certain products such as food preservatives poses challenges due to safety limits and marked organoleptic effects. Encapsulation of terpenes in nano or microstructured systems has been employed as a common approach to develop terpene formulations with improved chemical stability, shelf-life, additionally opening up the possibility to control terpene release [4,5,6,7,8].

We have developed methods using yeast particles (YPs) to efficiently encapsulate high levels of terpenes. YPs are 3–5 µm hollow and porous microspheres, a byproduct of the food grade Baker’s yeast (*Saccharomyces cerevisae*) extract manufacturing process. We have used YPs for the encapsulation of a broad range of molecules for drug delivery and agricultural applications [9,10,11,12,13,14,15]. Our first-generation approach to load terpenes inside the hydrophobic cavity of YPs is based on passive diffusion of terpenes through the porous microsphere cell walls without the need for surfactants or alcohols. Short-term sustained terpene release from YPs is the reverse process and is a function of terpene water solubility. This approach has been successfully implemented to develop and commercialize a YP-terpene based fungicide and nematicide for agricultural applications [16,17,18,19,20,21]. More recently, we have identified YP terpenes with broad-acting anthelmintic activity [22], which could lead to the development of formulations for oral terpene delivery for the treatment of gastrointestinal worm parasites and other infectious agents.

To develop better controlled terpene release, as well as to avoid limitations associated with terpene volatility and stability, we have developed a second generation YP terpene encapsulation approach using non-volatile, biodegradable pro-terpene compounds. The pro-terpene compounds are (1) solids at room temperature with a high melting point to avoid terpene loss due to the high volatility of terpenes, (2) water insoluble to avoid premature release from aqueous-based YP compositions, (3) stable at neutral pH, and (4) susceptible to chemical (pH) or enzymatic hydrolysis of a biodegradable linker providing for controlled release. This approach was first demonstrated for the YP encapsulation of a pro-carvacrol compound [23]. The YP pro-carvacrol was shown to have similar in vitro antibacterial properties as YP carvacrol, but with the additional benefits of improved stability and controlled, sustained carvacrol release from YPs.

In this article, we report the use of the YP pro-terpene encapsulation approach of four terpenes: carvacrol, eugenol, thymol, and geraniol and expanded testing against a wider range of applications. The YP terpene and YP pro-terpene compositions were characterized for controlled terpene release and screened in vitro for antibacterial, antifungal, and anthelmintic activity in model systems. The results show the potential broad range of applications of YP terpenes and YP pro-terpenes, and the advantage of YP pro-terpene encapsulation to improve the formulation stability and to control terpene release.

## 2. Materials and Methods

### 2.1. Materials

Yeast Particles (YPs) were purchased from Biorigin (Louisville, KY, USA). Terpenes (carvacrol, eugenol, thymol, and geraniol) were procured from Penta Manufacturing (Livingston, NJ, USA). All reagents and solvents for synthesis and HPLC analysis were obtained from Fisher Scientific (Waltham, MA, USA) or Sigma Aldrich (St. Louis, MO, USA). Lysogeny broth (LB) was purchased from Sigma Aldrich and yeast peptone dextrone (YPD) was prepared from Difco^TM^ yeast extract, Difco^TM^ Bacto peptone and dextrose (all materials obtained from Fisher Scientific) at a composition of 1% yeast extract, 2% peptone and 2% dextrose. Reagents for worm culture medium were purchased from Gibco (Gaithersburg, MO, USA).

### 2.2. Methods

#### 2.2.1. Synthesis of Pro-Terpene Compounds

Pro-terpenes were synthesized from the reaction of the parent terpene compound and ethylenediaminetetracetic acid (EDTA) dianhydride based on a previously described procedure for phenolic terpenes [23,24]. Briefly, terpene (18 mmol) and triethylamine (TEA, 64 mmol) were dissolved in 75 mL of anhydrous tetrahydrofuran (THF). EDTA dianhydride (9 mmol) was added slowly to the THF solution. The reaction mixture was stirred under nitrogen, at room temperature, overnight. The mixture was diluted in 500 mL of water and concentrated HCl was added to immediately acidify to pH 2. The precipitated product was filtered, washed with water, and dried under vacuum.

Pro-carvacrol–Yield: 71%; off-white powder; ^1^H-NMR (DMSO-d_6_, 500 MHz): δ ppm, 7.2 (d, 2H, Ar-H); 7.04 (d, 2H, Ar-H); 6.9 (s, 2H, Ar-H); 3.9 (s, 4H, CH_2_); 3.67 (s, 4H, CH_2_); 3.03 (s, 4H, CH_2_); 2.89 (m, 2H, CH); 2.05 (s, 6H, CH_3_); 1.1 (d, 12H, CH_3_)

Pro-eugenol–Yield: 81%; off-white powder; ^1^H NMR (DMSO-d_6_, 500 MHz): δ ppm, 6.9–7.0 (m, 4H, Ar-H); 6.7–6.8 (d, 2H, Ar-H); 6.0 (m, 2H, CH); 5.0–5.1 (m, 4H, CH_2_); 3.8 (s, 4H, CH_2_); 3.7 (s, 6H, CH_3_); 3.5 (s, 4H, CH_2_); 3.4 (d, 4H, CH_2_); 2.9 (s, 4H, CH_2_)

Pro-thymol–Yield: 60%; off-white powder; ^1^H NMR (DMSO-d_6_, 500 MHz): δ ppm, 7.2 (d, 2H, Ar-H), 7.05 (d, 2H, Ar-H); 6.85 (s, 2H, Ar-H); 3.9 (s, 4H, CH_2_); 3.6 (s, 4H, CH_2_); 2.9 (s, 4H, CH_2_); 2.8 (m, 2H, CH); 2.2 (s, 6H, CH_3_); 1.1 (d, 12 H, CH_3_)

Pro-geraniol–Yield: 27%; off-white powder; ^1^H NMR (DMSO-d_6_, 500 MHz): δ ppm, 5.35 (m, 2H, CH); 5.1 (m, 2H, CH); 4.6 (m, 4H, CH_2_); 3.95 (s, 4H, CH_2_); 3.6 (s, 4H, CH_2_); 3.1 (s, 4H, CH_2_); 2.0–2.1 (m, 8H, CH_2_); 1.65 (m, 12 H, CH_3_); 1.55 (s, 6H, CH_3_)

#### 2.2.2. YP Loading of Terpenes (YP Terpene)

Dry YPs were mixed with 0.5 µL water per mg YP. Then, terpene was absorbed into YPs by adding 1 mg terpene per mg YP and incubated at room temperature for 18–24 h for samples containing carvacrol, geraniol or eugenol, and at 65 °C for 18–24 h for samples containing thymol.

#### 2.2.3. YP Loading of Pro-Terpenes (YP Pro-Terpene)

Dry YPs were mixed with 0.5 µL water per mg YP. Then, pro-terpene was absorbed into YPs by swelling the particles with a solution of pro-terpene in DMSO (2.5 µL/mg YP). The samples were incubated at room temperature for 18–24 h to complete the loading. The YP pro-terpene was then lyophilized, and the loading process was repeated until the target concentration of encapsulated pro-terpene was achieved. YP pro-terpene samples contained 1.78–1.85 mg pro-terpene per mg YP to yield 1 mg terpene/mg YP upon pro-terpene hydrolysis.

#### 2.2.4. Characterization of Terpene and Pro-Terpene Loading Efficiency

Samples were stained with Nile red to qualitatively assess loading by the fluorescence microscopy of the encapsulated terpene or pro-terpene Nile red complex. YP terpene and YP pro-terpene samples were suspended in water at a concentration of 10 mg YP/mL. The samples were centrifuged to collect excess liquid (free terpene or pro-terpene). The supernatants and YP pellets were incubated in 0.1 M sodium carbonate buffer (pH 10) for 3 h to hydrolyze pro-terpene and then the samples were diluted with methanol to quantify free (supernatant fraction) and YP encapsulated (pellet fraction) terpene. Terpenes were quantified by HPLC [22] operated with 32 Karat^TM^ software version 7.0 (Beckman Coulter, Inc, Brea, CA, USA), using a Waters Symmetry^®^ C18 column (3.5 µm, 4.6 × 150 mm) with acetonitrile:water 70:30 as mobile phase, flow rate at 1 mL/min, injection volume of 10 µL, and terpene detection at 254 nm. This isocratic HPLC method allows for the detection of single terpene samples with the following retention times: 3.35 min (carvacrol), 2.73 min (eugenol), 3.35 min (geraniol), and 3.42 min (thymol). The quantification of terpenes was done by measuring the peak area and interpolating the concentration using a calibration curve obtained with terpene standards.

#### 2.2.5. Terpene Release from YPs

YP terpene and YP pro-terpene samples were suspended in phosphate buffer saline (PBS, pH 7) at a concentration of 1 mg YP/mL (=1 mg terpene/mL) and incubated at 37 °C. Aliquots were collected at predetermined times, centrifuged and the supernatant was collected to quantify terpene released from the particles by HPLC.

#### 2.2.6. Antimicrobial Activity Assays in Model Bacterial and Fungal Organisms

The antimicrobial activity of YP terpene and YP pro-terpene was evaluated using a modified microplate assay published procedure [25]. Samples of YP terpene and YP pro-terpene were suspended in 100 µL of growth medium (LB was used in antibacterial assays and YPD in antifungal assays) and added to the first row (Row A) of a 96-well plate (all wells in the 96-well plate contain additional 100 µL medium). Serial dilution (1:1) was performed by transferring 100 µL from Row A to Row B, etc., and finally removing 100 µL from Row H. Diluted *Escherichia coli* Top10 (Invitrogen, Carlsbad CA) or *Saccharomyces cerevisae* Cry1 [26] cells (100 µL, 10^6^ cells/mL) were added to all wells of the plate. Initial (*t* = 0) and final (*t* = 16 h, 37 °C) absorbance readings were taken at 650 nm. The minimum inhibitory concentration (MIC) was determined as the concentration that inhibits bacterial or fungal growth by more than 75%.

#### 2.2.7. Antimicrobial Assay with Fractionated Samples

YP pro-carvacrol samples (10 mg carvacrol/mL) were incubated 18 h at 37 °C in fresh LB and LB collected from *E. coli* culture (spent LB). The samples were centrifuged to collect the supernatant and YP pellet fractions; an aliquot of the supernatant was used to quantify released carvacrol by HPLC. The pellet fractions were suspended in water and an aliquot was used for HPLC analysis. Both supernatant and pellet fractions used for HPLC analysis were incubated in 0.1 M carbonate buffer (pH 10) for 3 h to hydrolyze pro-terpene and then diluted with methanol (final composition of 90% methanol) to solubilize terpene. The remaining supernatant and pellet samples were evaluated for antibacterial activity on *E. coli*, as described above.

#### 2.2.8. Simulated Digestion Assay

YP terpene and YP pro-terpene samples were suspended in simulated gastric fluid (SGF) [27] containing 3.2 mg pepsin/mL at a concentration of 1 mg terpene/mL. The samples were incubated for 1 h at 37 °C, centrifuged and the SGF supernatant was collected. The pellet was suspended in simulated intestinal fluid (SIF) [28] containing 10 mg pancreatin/mL, incubated at 37 °C for 1 h, and centrifuged to collect SIF supernatant. The pellet was suspended again in SIF with fresh pancreatin, and incubated at 37 °C for 1 h. Terpene released from YPs was quantified in all supernatants by HPLC.

#### 2.2.9. Nematode Extract Assay

*Ascaris suum* 4th stage larvae (350 mg) were isolated from pig intestines between 14–21 days after inoculation [29] and were immersed in PBS (pH 7) or 0.1 M sodium acetate buffer (pH 5) and sonicated with a microtip sonicator probe at maximum power multiple times for 30 s until the worm was disintegrated. Samples were kept on ice during sonication. Total protein in worm extracts was quantified using the bicinchoninic acid protein assay kit (ThermoFisher Scientific, Waltham, MA, USA) with bovine serum albumin control (10.3 ± 1.7 mg protein/mL in worm extract pH 7, 9.2 ± 0.5 mg protein/mL in worm extract pH 5). YP pro-carvacrol samples were suspended in worm extract at a target carvacrol concentration of 20 mg/mL for 24 h at 37 °C. Control YP pro-carvacrol samples were incubated in PBS, acetate buffer, and 0.1 M carbonate buffer pH 10. Samples were diluted 1:1 with methanol, centrifuged, the supernatant was collected and the released carvacrol was quantified by HPLC. Supernatants and YP pro-carvacrol pellets were evaluated for antimicrobial activity on *E. coli*.

#### 2.2.10. Larval Development (Egg-to-Larvae (E2L)) Assay

An E2L assay was used to measure the effects of carvacrol and pro-carvacrol on the development of cyathostomin larvae from eggs to third-stage larvae (L3) [30]. Cyathostomin eggs were collected from the feces of an equine herd [31]. Approximately 60 nematode eggs were added to each well of a 96-well plate using a repeat dispensing pipette. YP samples (10 µL of each working solution) were impregnated into 90 µL of S medium with *E. coli* OP50. Control wells received 10 µL of water only. The plates were incubated for 7 days at 28 °C, larvae were then killed using Lugol’s iodine, and the number of fully grown infective L3 were counted in each well. Each concentration-response experiment consisted of triplicate wells.

#### 2.2.11. Adult Worm In Vitro Screening

*Ancylostoma ceylanicum* worms were maintained in golden Syrian hamsters, as previously described [32]. *Trichuris muris* parasites were maintained in STAT6-/-mice [33]. Adult worms were harvested from infected rodents and washed using prewarmed medium (RPMI 1640 with 25 mM HEPES (pH 7.2) and antimicrobials (100 U/mL penicillin, 100 µg/mL streptomycin, fungizone (10 µg/mL for *A. ceylanicum*, 2.5 µg/mL for *T. muris*)). Worms were manually picked into the wells of the 48-well screening plate (1 worm per well) containing 250 µL RPMI per well. As serum is incompatible with the assays with YP-terpenes, it was left out. YP terpene and YP pro-terpene samples were evaluated at a concentration of 333 µg terpene/mL [22]. Assay plates were incubated at 37 °C and 5% CO_2_. Terpene activity was determined by motility of adult worms measured with an in-house assembled Worminator [34]. The Worminator consists of a dark field illuminator, plate holder and video camera placed under the assay plate. Worm motility was recorded using the “WormAssay” software [34], measuring the average motility in each well based on pixel displacement between frames over a given time. Data is expressed as mean motility units and percent inhibition of motility was calculated relative to the mean motility units of control worms (worms incubated with media).

## 3. Results

### 3.1. Preparation and Characterization of Yeast Particle Encapsulated Terpenes and Pro-Terpenes

#### 3.1.1. Synthesis of Pro-Terpenes

To improve the stability of YP-terpenes and to better control sustained terpene release, we designed pro-terpene compounds with the following properties: (1) solid at room temperature to prevent terpene loss due to high volatility of terpene compounds, (2) water insoluble to avoid premature terpene release and loss in diluted samples, (3) stable at neutral pH, and (4) susceptible to chemical or enzymatic hydrolysis of a biodegradable linker providing for controlled terpene release.

**Figure 1:**
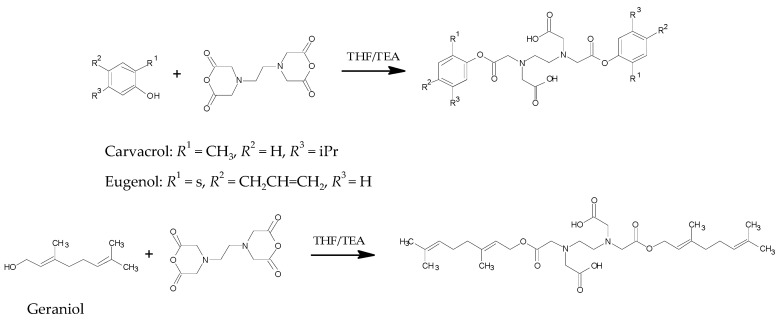
Synthesis of pro-terpenes.

Pro-terpenes were synthesized via ring-opening transesterification of EDTA dianhydride with the hydroxyl group of terpenes (carvacrol, eugenol, thymol, and geraniol) in the presence of triethylamine (TEA) to yield diacids of terpenes with ester biodegradable bonds, as depicted in Figure 1. The pro-terpenes have high melting points compared to terpenes (three of the terpenes are liquid at room temperature) and are practically insoluble in water (Table 1). The high melting point, poor water solubility, and biodegradable linkers make these pro-terpenes suitable candidates for the development of stable YP pro-terpene formulations with controlled terpene release.

#### 3.1.2. Yeast Particle Encapsulation of Terpenes and Pro-Terpenes

**Figure 2:**
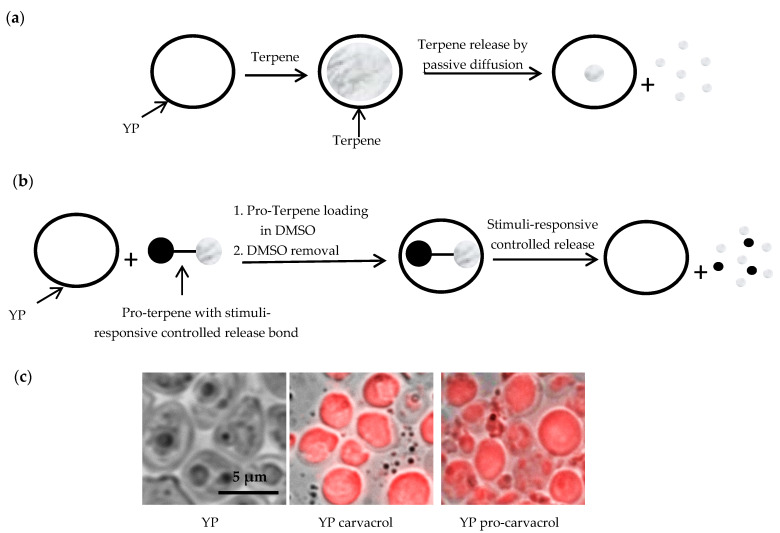
Schematics of (**a**) diffusion-controlled terpene loading in YPs and terpene release, (**b**) pro-terpene loading in YPs and stimuli-controlled terpene release and (**c**) microscopy images of Nile red stained YP control and YPs loaded with carvacrol and pro-carvacrol.

YPs are 3–5 µm, hollow and porous microparticles derived from Baker’s yeast. The porous cell wall structure makes these particles excellent absorbent materials, and payloads can be loaded from aqueous and some organic solutions with high payload loading capacity and efficiency in the large hollow cavity of the particles. Our first-generation terpene encapsulation approach is depicted in Figure 2a and is based on the loading of terpenes inside the hydrophobic cavity of YPs by the passive diffusion of the payload through the porous cell walls in an aqueous suspension of YPs. High terpene loading (1:1 *w*/*w* terpene:YP for materials reported in this publication and up to 3:1 *w*/*w* (unpublished results)) is achieved with this method. Terpene release from YPs is based on passive diffusion out of the particles and is a function of terpene water solubility with complete terpene release in minutes to a few hours [22].

A second-generation approach was developed to better control terpene release from YP encapsulated pro-terpenes (Figure 2b). The pro-terpenes are highly soluble in dimethylsulfoxide (DMSO), a suitable solvent for payload loading in YPs that is removed by lyophilization without loss of pro-terpene. Encapsulation of terpene and pro-terpenes in YPs was assessed qualitatively by microscopy and quantitatively by HPLC. Nile red dye was used to stain terpenes and pro-terpenes to visualize payload encapsulation, as shown in Figure 2c for YP samples containing carvacrol. The HPLC results showed that terpenes and pro-terpenes were encapsulated with >95% efficiency at a target loading ratio of 1:1 terpene:YP or 1.85–1.90:1 pro-terpene:YP (to yield a 1:1 terpene:YP and 0.85–0.9:1 EDTA:YP ratio).

#### 3.1.3. Terpene Release from YP Terpene and YP Pro-Terpene Samples

Terpene release from YP terpene is based on passive diffusion out of the particles and is a function of terpene water solubility. The four terpenes evaluated in this study have similar water solubility (~1 mg/mL) and terpenes are rapidly released from YPs (e.g., >90% carvacrol released at 1 h) upon dilution at concentrations equal or less than 1 mg/mL [22].

The pro-terpenes are water insoluble and their release from YPs is dependent on pH or enzymatic hydrolysis of the ester bonds. We previously showed [23] that the kinetics of pro-terpene hydrolysis is pH dependent with the ester bonds of the pro-terpenes being susceptible to fast hydrolysis in basic pH (>50% pro-carvacrol hydrolyzed within 30 min at pH 10) and increased stability at lower pH values (it took 4 days at pH 7, 21 days at pH 5, and more than 2 months at pH 1.5 to reach 50% hydrolysis of pro-carvacrol) [23].

**Figure 3:**
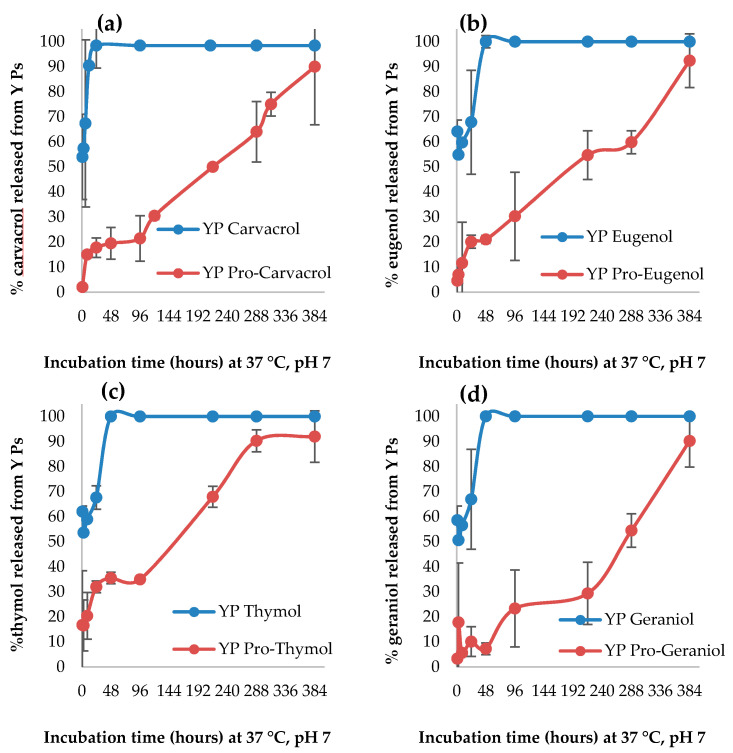
Kinetics of terpene release from YP terpene and YP pro-terpene containing (**a**) carvacrol, (**b**) eugenol, (**c**) thymol, and (**d**) geraniol. Samples were incubated in 0.1 M phosphate buffer saline (PBS, pH 7) at 37 °C at a concentration of 1 mg terpene/mL.

The YP terpene and YP pro-terpene compositions were evaluated for terpene release in phosphate buffer saline (PBS, pH 7) at 37 °C. The results in Figure 3 clearly show that YP pro-terpenes have greater pH 7 stability than YP terpenes. All YP terpene samples rapidly released their payload in PBS upon dilution of the sample to 1 mg terpene/mL. YP pro-terpenes resuspended in PBS at the same concentration showed a small burst release (<20%), followed by slow hydrolysis and sustained terpene release over two weeks to achieve complete control, and sustained terpene release from YPs.

**Figure 4:**
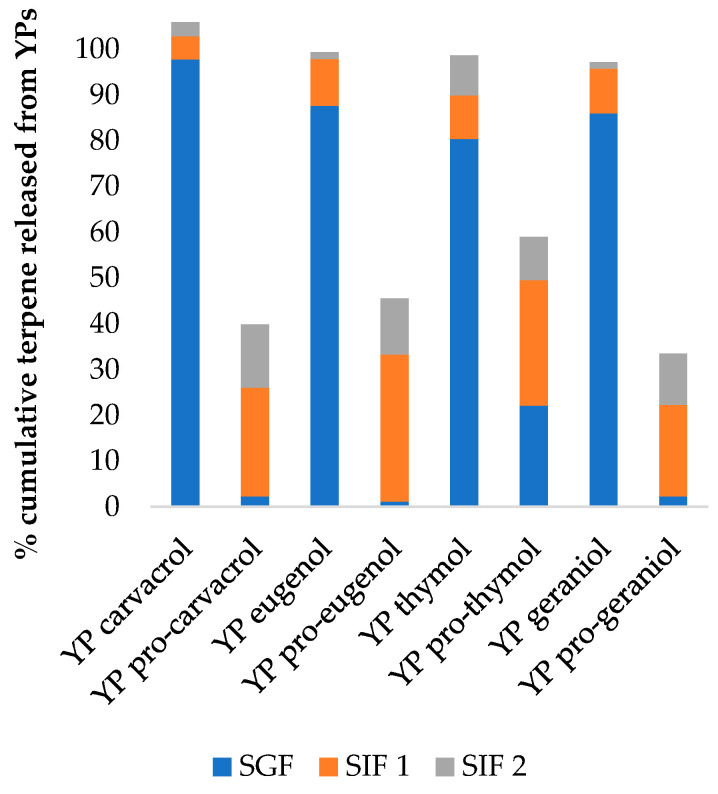
Cumulative terpene released from YPs after 1 h incubation in simulated gastric fluid (SGF) containing pepsin and after two 1 h incubations in fresh simulated intestinal fluid (SIF) with pancreatin.

Next, we evaluated YP terpenes and YP pro-terpenes samples for stability during simulated digestion (Figure 4). Samples were first incubated in simulated gastric fluid (SGF) containing pepsin, followed by incubation in simulated intestinal fluid (SIF) containing pancreatin.

Terpenes are released from YP terpenes in SGF, primarily due to terpene diffusion out of the particles. YP pro-terpenes are stable in SGF due the pro-terpenes water insolubility, slow hydrolysis at pH < 2, and non-susceptibility to degradation by pepsin (pepsin is an endopeptidase that breaks amide peptide bonds). Terpenes were partially released from YP pro-terpenes in SIF due to the presence of lipases in pancreatin that hydrolyze ester linkages. Pancreatin activity in simulated digestion is lost within 1 h. To demonstrate the effect of enzyme activity on YP pro-terpene hydrolysis and release, samples were subjected to two sequential 1 h incubations in fresh SIF + pancreatin.

### 3.2. Biological Activity of YP Pro-Terpenes

The biological activity of YP pro-terpenes was evaluated against different model organisms to demonstrate that YP encapsulated pro-terpenes retain the broad-spectrum anti-pathogen effects (antibacterial, antifungal, and anthelmintic) of free terpenes and YP terpenes.

Antimicrobial activity of YP pro-terpenes: YP pro-terpene samples were tested for antibacterial activity against *E. coli.* Table 2 shows the minimum inhibitory concentrations (MICs) of YP samples and controls. Empty YPs have no antimicrobial effect on *E. coli.* For the three terpenes evaluated on *E. coli*, the free terpene, YP terpene and YP pro-terpene samples show similar antibacterial activity. Hydrolysis of pro-terpenes generates EDTA as byproduct and a control sample of YP+EDTA containing the same amount of EDTA generated from pro-terpene hydrolysis was evaluated to confirm that the antimicrobial effect of YP pro-terpene samples was due to the terpene released from the particles and not from the EDTA byproduct.

The antibacterial effect against *E. coli* is due to bacterial absorption of terpene present in the LB medium added as free terpene or terpene released from YP terpenes (all MICs are below the maximum solubility of terpenes in water). The kinetics of pro-terpene hydrolysis at neutral pH (pH of LB = 6.8) make it unlikely to achieve high enough terpene release from YP pro-terpene samples during the 18 h incubation required for this experiment. Additionally, the pro-terpenes are insoluble in LB medium and YPs are not internalized by *E. coli*; therefore, it is not possible for pro-terpene hydrolysis to occur inside the bacteria. We hypothesized that YP pro-terpenes are hydrolyzed by esterases secreted from *E. coli* into the LB medium. Samples of YP pro-carvacrol were incubated for 18 h in fresh LB or in LB bacterial cell-free spent media collected after *E. coli* culture (LB spent media). The samples were centrifuged to collect the supernatant and YP pellet fractions; carvacrol generated from pro-terpene hydrolysis was quantified in supernatants by HPLC, and the samples were added to *E. coli* to evaluate the antibacterial activity of each fraction (Table 3). The YP pro-carvacrol samples incubated in fresh LB medium released <5% of carvacrol. Samples incubated in spent media released 35 ± 6% carvacrol into the supernatant confirming that pro-terpenes are hydrolyzed by enzymes secreted from *E. coli* into the LB medium. The HPLC analysis of the pellet fractions showed that carvacrol retained in the pellet was in the form of pro-carvacrol for both samples incubated in fresh or spent media. The HPLC results were used to correct carvacrol concentration in supernatant and pellet fractions added to *E. coli*. The results in Table 3 show that only the pellet fraction was active for the sample incubated in fresh LB medium and both fractions collected from spent LB medium were active.

Next, we evaluated YP pro-terpenes for antifungal activity against *S. cerevisae*. The results in Table 4 show that empty YP and YP+EDTA are not active against *S. cerevisae* and the three terpenes evaluated in this assay show similar MIC values for free terpene, YP terpene, and YP pro-terpene samples. The activity of YP pro-terpene sample is likely due to a similar effect shown with *E. coli*, with terpene release from YPs upon hydrolysis of pro-terpene induced by esterases secreted by fungi.

Anthelmintic activity of YP pro-terpenes: We recently reported the testing of 17 YP terpenes as broad-acting anthelmintics [22]. YP terpenes or subsets of them were active against hookworms (*Ancylostoma ceylanicum* and *Nippostrongylus brasiliensis*) and whipworm (*Trichuris muria*), and overcame albendazole-resistant *Caenorhabditis elegans*. YP encapsulation provides an approach that could lead to the development of anthelmintic terpene formulations for oral delivery. The new YP encapsulation approach using pro-terpenes with stimuli-controlled terpene release could provide materials that overcome fast terpene release and absorption in the stomach.

First, we evaluated if YP pro-terpenes are susceptible to hydrolysis by enzymes in a nematode (*Ascaris suum*) extract. YP pro-carvacrol samples were incubated in *Ascaris* extract at pH 7 and pH 5. Control samples were incubated in buffer only including a control in 0.1 M carbonate buffer (pH 10) for complete pro-carvacrol hydrolysis and carvacrol release from YP. The samples were centrifuged to collect the supernatant and YP pellet fractions, carvacrol was quantified in supernatants, and both fractions were evaluated for antimicrobial activity on *E. coli*. The results in Table 5 show that YP pro-carvacrol was susceptible to hydrolysis by esterases in the *Ascaris* extract with 5–6-fold higher carvacrol release in the *Ascaris* extract compared to buffer controls. There was antimicrobial activity from terpene present in both supernatant and pellet fractions of samples incubated in *Ascaris* extract, but only the pellet fraction of samples incubated in pH 7 and pH 5 buffers is active. The control sample in pH 10 buffer (no worm extract) showed the expected complete carvacrol release and antibacterial activity only in the supernatant fraction.

Next, the biological activity of YP pro-terpenes and YP terpenes was assessed in three in vitro nematode assays: (1) development of the horse parasite cyathostomin from egg to third-stage larvae [30], (2) toxicity in adult hookworm (*A. ceylanicum*) and (3) toxicity in adult whipworm (*T. muris*).

**Figure 5:**
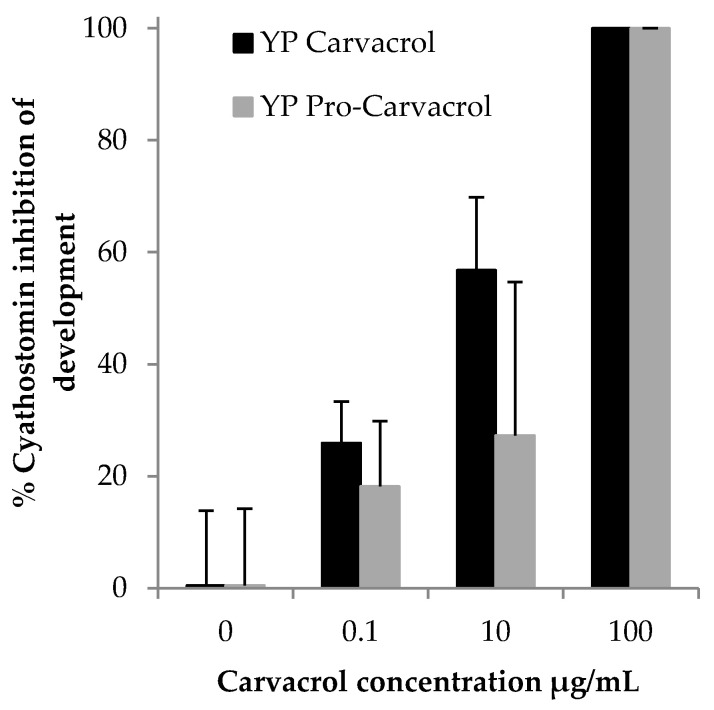
In vitro activity of carvacrol samples in cyathostomin egg-to-larvae (E2L) assay.

YP pro-carvacrol and YP carvacrol were evaluated in the cyathostomin E2L assay with samples showing similar dose-response activity (Figure 5) completely inhibiting E2L development at a concentration of 100 µg carvacrol/mL. A control of YP+EDTA also had some toxicity (~40–45% inhibition of larvae development, data not shown) at the highest EDTA concentration equivalent to the expected amount of EDTA generated from the hydrolysis of YP pro-carvacrol at 100 µg/mL. The long incubation period (seven days) required for the Cyathostomin E2L assay likely increased the impact of EDTA complexation of metal ions critical in processes of larvae development.

**Figure 6:**
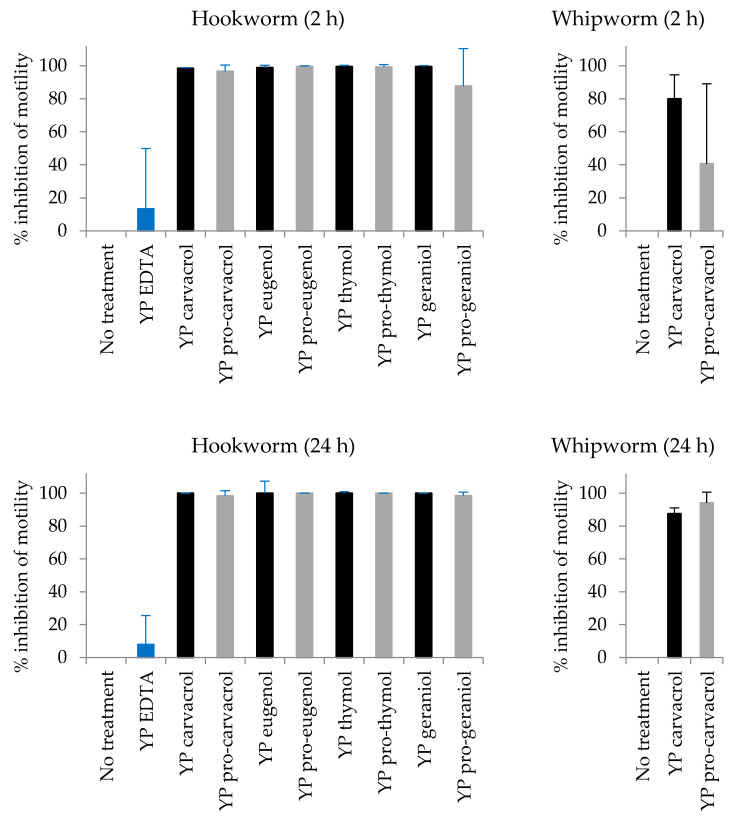
Inhibition of adult hookworm and whipworm motility by YP terpenes and YP pro-terpenes after 2 and 24 h incubation (results are average of eight worms per treatment). Percent inhibition of motility was calculated relative to the mean motility units of control worms (no treatment).

YP pro-terpene and YP terpene samples were evaluated at a concentration of 333 µg terpene/mL in the adult hookworm and whipworm assays. We previously identified all four terpenes to have a fast-acting effect on hookworm at the selected terpene concentration of 333 µg /mL [22]. We also demonstrated that hookworms readily ingest YPs which leads to two possible mechanisms of terpene entry: (1) hookworm ingestion of terpene released from YPs or (2) hookworm ingestion of YP terpene or YP pro-terpene and subsequent terpene release inside the worms. The results in Figure 6 show that all samples were active against hookworm after 2-h and 24 h incubation and YP+EDTA control was non-toxic.

YP pro-carvacrol and YP carvacrol samples were evaluated for in vitro activity in the whipworm assay at a concentration of 333 µg/mL. Unlike hookworms, whipworms do not ingest YPs and therefore toxicity of YP terpenes in whipworm only occurs due to whipworm absorption of terpene released from YPs into the whipworm assay media [22]. The results in Figure 6 show >70% inhibition of whipworm motility after 2 h incubation with YP carvacrol due to fast release of carvacrol from YP carvacrol, but the YP pro-carvacrol sample is less active after 2 h incubation. This reduced activity after 2-h is expected as carvacrol release from YP pro-carvacrol requires hydrolysis of pro-carvacrol mediated by esterases secreted by whipworm into the media, followed by carvacrol diffusion from YPs. Both YP carvacrol and YP pro-carvacrol showed similar inhibition of whipworm motility after 24 h incubation.

## 4. Discussion

Terpenes are natural products of great commercial interest due to their wide array of functional properties. Microencapsulation of terpenes in some products is challenging due to their high volatility and susceptibility to degradation. We previously developed methods to use yeast particles for the encapsulation of terpenes without the need for alcohols or surfactants. These YP terpenes have been shown to exhibit broad anthelmintic activity [22] and two YP terpene-based products have been developed and commercialized as a fungicide and nematicide for agricultural applications [16,17,18,19,20,21]. The goals of developing a second generation of YP terpene materials were to improve terpene encapsulation stability and to provide for stimuli-responsive controlled terpene release. This new approach employs pro-terpene compounds that are (1) solid at room temperature to avoid loss due to the high volatility of terpenes, (2) water-insoluble to prevent premature release from YPs, (3) stable at neutral pH, and (4) contain a stimuli-controlled (pH, enzyme) biodegradable bond for controlled terpene release.

High encapsulation efficiency (>95%) of both pro-terpenes or terpenes in YPs was achieved at a target terpene:YP weight ratio of 1:1. Terpene release from YP terpenes is dependent on the diffusion of terpene from the particles and is a function of terpene solubility with complete release in minutes to a few hours upon dilution of YP terpene below its maximum solubility in water. Terpene release from YP pro-terpenes is a function of pro-terpene hydrolysis in response to an external stimulus, followed by terpene diffusion from YPs. Terpene encapsulation stability is improved using pro-terpenes, extending the release of terpene at pH 7 from a few hours (YP terpene) up to two weeks (YP pro-terpenes).

The YP pro-terpene and YP terpene samples showed similar biological activity in antibacterial, antifungal and anthelmintic in vitro assays. The activity of YP pro-terpene in these assays is mediated by the presence of esterases to induce hydrolysis of the pro-terpene and subsequent terpene release from YPs. The enhanced stability and controlled release of YP pro-terpenes could allow for developing terpene formulations for applications such as (1) environmental biocontrol agent applied directly to contaminated soil or a pass through in feed to reduce transmission of gastrointestinal parasitic nematodes by targeting developing/infectious larvae of parasites that infect livestock and humans in the third larval stage (e.g., cyathostomins, *Haemonchus*, *Ostertagia*, hookworms), (2) formulations for oral delivery of terpenes for targeting of internal gastrointestinal parasites, and (3) controlled-release of terpenes as food fragances and flavors, or as antimicrobial agents in food preservation. Future work will focus on the in vivo evaluation of YP pro-terpenes.

This new approach of payload encapsulation in YPs using pro-terpenes can be expanded to a broad range of small drug molecules that are difficult to trap using previously developed yeast particle drug encapsulation methods. We are currently investigating this approach to stably generate encapsulated yeast particle pro-drugs with stimuli-controlled drug release for a broad range of payload molecules such as antibacterials (e.g., isoniazid, oxazolidinones, and cycloserine), anti-inflammatories (e.g., naproxen, ibuprofen), and chemotherapeutics (e.g., doxorubicin).

## 5. Conclusions

Yeast particles can be used for the encapsulation of scaffolded terpene compounds containing a biodegradable linker to control terpene release. These YP pro-terpene samples exhibit the same loading capacity as YP terpenes, and with additional benefits of improved stability and control over terpene release in response to pH or esterase induced hydrolysis of the pro-terpene compound. Both YP terpenes and YP pro-terpenes exhibit biological activity in antimicrobial and anthelmintic assays.

## 6. Patents

Yeast Cell Wall Particle Encapsulation of Biodegradable Pro-Payloads. G.R. Ostroff and E. R. Soto. US Patent App. 16/981,072, 2021. 28 January 2021.

## Figures and Tables

**Table 1:** Melting point and solubility in water of terpenes and pro-terpenes.

Compound	Melting Point (°C)	Solubility in Water
Carvacrol ^1^	3.5	1.25 mg/mL
Pro-carvacrol ^2^	156–158	Insoluble
Eugenol ^1^	−9.2	1.44 mg/mL
Pro-eugenol ^2^	167–169	<20 µg/mL
Thymol ^1^	51.5	0.90 mg/mL
Pro-thymol ^2^	135–138	Insoluble
Geraniol ^1^	−15	0.1 mg/mL
Pro-geraniol ^2^	Decomposes above 145	Insoluble

^1^ Terpene data from PubChem [35]. ^2^ Pro-terpene data determined experimentally

**Table 2:** In vitro antibacterial activity on *E. coli* of free terpene, YP terpene and YP pro-terpene samples.

Sample		MIC 75% (Average of *n* = 4)
Negative controls	Empty YP	Not active
	YP + EDTA	4244 ± 1400 µg EDTA/mL
Carvacrol samples	Carvacrol	625 ± 0 µg carvacrol/mL
	YP carvacrol	677 ± 45 µg carvacrol/mL
	YP pro-carvacrol	625 ± 0 µg carvacrol/mL
Eugenol samples	Eugenol	938 ± 361 µg eugenol/mL
	YP eugenol	312 ± 0 µg eugenol/mL
	YP pro-eugenol	625 ± 442 µg eugenol/mL
Geraniol samples	Geraniol	1094 ± 313 µg geraniol/mL
	YP geraniol	781 ± 312 µg geraniol/mL
	YP pro-geraniol	1094 ± 312 µg geraniol/mL

**Table 3:** In vitro antibacterial activity on *E. coli* of YP pro-carvacrol sample before and after incubation in LB spent media.

LB	Fraction	MIC 75% (µg Carvacrol/mL, Average of *n* = 3)
Fresh	Supernatant	Not active
	Pellet	521 ± 180
Spent	Supernatant	500 ± 216
	Pellet	729 ± 252

**Table 4:** In vitro antifungal activity on *Saccharomyces cerevisae* of free terpene, YP terpene and YP pro-terpene samples.

Sample		MIC 75% (Average of *n* = 3)
Negative controls	Empty YP	Not active
	YP + EDTA	Not active
Eugenol samples	Eugenol	703 ± 773 g eugenol/mL
	YP eugenol	390 ± 193 µg eugenol/mL
	YP pro-eugenol	781 ± 362 µg eugenol/mL
Thymol samples	Thymol	312 ± 110 µg thymol/mL
	YP thymol	1250 ± 442 µg thymol/mL
	YP pro-thymol	781 ± 312 µg thymol/mL
Geraniol samples	Geraniol	703 ± 773 µg geraniol/mL
	YP geraniol	312 ± 221 µg geraniol/mL
	YP pro-geraniol	260 ± 90 µg geraniol/mL

**Table 5:** Hydrolysis of YP pro-carvacrol in *Ascaris suum* extract and antibacterial activity on the *E. coli* of the supernatant containing carvacrol released from the YPs and YP pellet fraction containing residual encapsulated pro-carvacrol.

Buffer ± *Ascaris* Extract	% Carvacrol Released from YPs after 24 h Incubation	MIC 75% (µg carvacrol/mL, Average of *n* = 3)	
		Supernatant	Pellet
PBS (pH 7)	4.5 ± 1.8	Not active	597
*Ascaris* extract in PBS (pH 7)	27.9 ± 6.0	291 ± 101	451 ± 0
Acetate buffer (pH 5)	6.5 ± 0.9	325	584
*Ascaris* extract in acetate buffer (pH 5)	37.3 ± 9.5	155 ± 67	261 ± 113
Carbonate buffer (pH 10)	95.6 ± 1.2	598	Not active

## Data Availability

The data presented in this study are available upon request from the corresponding author.

## References

[1] Cowan M.M. (1999). Plant products as antimicrobial agents. Clin. Microbiol. Rev..

[2] Falleh H., Ben Jemaa M., Saada M., Ksouri R. (2020). Essential oils: A promising eco-friendly food preservative. Food Chem..

[3] Ben Salha G., Abderrabba M., Labidi J. (2019). A status review of terpenes and their separation methods. Rev. Chem. Eng..

[4] Bakry A.M., Abbas S., Ali B., Majeed H., Abouelwafa M.Y., Mousa A., Liang L. (2016). Microencapsulation of Oils: A Comprehensive Review of Benefits, Techniques, and Applications. Compr. Rev. Food Sci. Food Saf..

[5] Calo J.R., Crandall P.G., O’Bryan C.A., Ricke S.C. (2015). Essential oils as antimicrobials in food systems—A review. Food Control.

[6] Saifullah M., Shishir M.R.I., Ferdowsi R., Tanver Rahman M.R., Van Vuong Q. (2019). Micro and nano encapsulation, retention and controlled release of flavor and aroma compounds: A critical review. Trends Food Sci. Technol..

[7] Bhalerao Y.P., Wagh S.J. (2018). A Review on thymol encapsulation and its controlled release through biodegradable polymer shells. Int. J. Pharm. Sci. Res..

[8] De Matos S.P., Teixeira H.F., De Lima A.A.N., Veiga-Junior V.F., Koester L.S. (2019). Essential oils and isolated terpenes in nanosystems designed for topical administration: A review. Biomolecules.

[9] Soto E.R., Ostroff G.R. (2008). Characterization of multilayered nanoparticles encapsulated in yeast cell wall particles for DNA. Bioconjug. Chem..

[10] Aouadi M., Tesz G.J., Nicoloro S.M., Wang M., Chouinard M., Soto E., Ostroff G.R., Czech M.P. (2009). Orally delivered siRNA targeting macrophage Map4k4 suppresses systemic inflammation. Nature.

[11] Soto E., Ostroff G. (2012). Glucan particles as carriers of nanoparticles for macrophage-targeted delivery. Nanomaterials for Biomedicine.

[12] Mirza Z., Soto E.R., Dikengil F., Levitz S.M., Ostroff G.R. (2017). Beta-glucan particles as vaccine adjuvant carriers. Vaccines for Invasive Fungal Infections.

[13] Soto E.R., O’Connell O., Dikengil F., Peters P.J., Clapham P.R., Ostroff G.R. (2016). Targeted Delivery of Glucan Particle Encapsulated Gallium Nanoparticles Inhibits HIV Growth in Human Macrophages. J. Drug Deliv..

[14] Hamza Z., El-Hashash M., Aly S., Hathout A., Soto E., Sabry B., Ostroff G. (2019). Preparation and characterization of yeast cell wall beta-glucan encapsulated humic acid nanoparticles as an enhanced aflatoxin B1 binder. Carbohydr. Polym..

[15] Huang H., Ostroff G.R., Lee C.K., Specht C.A., Levitz S.M. (2010). Robust Stimulation of Humoral and Cellular Immune Responses following Vaccination with Antigen-Loaded β-Glucan Particles. MBio..

[16] Franklin L., Ostroff G., Harman G. (2020). Terpene-Containing Compositions and Methods of Making and Using Them. U.S. Patent.

[17] Franklin L., Ostroff G. (2019). Compositions and Methods Comprising Terpenes or Terpene Mixtures Selected from Thymol, Eugenol, Geraniol, Citral and L-Carvone. U.S. Patent.

[18] Franklin L., Ostroff G. (2020). Compositions Containing a Hollow Glucan Particle or a Cell Wall Particle Encapsulating a Terpene Component, Methods of Making and Using Them. U.S. Patent.

[19] Franklin L., Ostroff G. (2018). Nematicidal Compositions and Methods of Using Them. U.S. Patent.

[20] Franklin L., Ostroff G. (2017). Nematicidal Compositions and Methods of Using Them. U.S. Patent.

[21] Franklin L., Ostroff G. (2016). Compositions and Methods Comprising Terpenes or Terpene Mixtures Selected from Thymol, Eugenol, Geraniol, Citral, and L-Carvone. U.S. Patent.

[22] Mirza Z., Soto E.R., Hu Y., Nguyen T.T., Koch D., Aroian R.V., Ostroff G.R. (2020). Anthelmintic activity of yeast particle-encapsulated terpenes. Molecules.

[23] Soto E., Rus F., Ostroff G.R. (2019). Yeast Cell Wall Particle Encapsulation of Pro-Terpene Payloads. Nanotech 2019 TechConnect Briefs. https://briefs.techconnect.org/papers/yeast-cell-wall-particle-encapsulation-of-pro-terpene-payloads/.

[24] Carbone-Howell A.L., Stebbins N.D., Uhrich K.E. (2014). Poly(anhydride-esters) comprised exclusively of naturally occurring antimicrobials and EDTA: Antioxidant and antibacterial activities. Biomacromolecules.

[25] Sultanbawa Y., Cusack A., Currie M., Davis C. (2009). An innovative microplate assay to facilitate the detection of antimicrobial activity in plant extracts. J. Rapid Methods Autom. Microbiol..

[26] Harley C.A., Tipper D.J. (1996). The role of charged residues in determining transmembrane protein insertion orientation in yeast. J. Biol. Chem..

[27] U.S. Pharmacopeia (2000). Simulated Gastric Fluid, TS. National Formulary No. 24/19.

[28] U.S. Pharmacopeia (1995). Simulated Intestinal Fluid.

[29] Urban J.F., Hu Y., Miller M.M., Scheib U., Yiu Y.Y., Aroian R.V. (2013). *Bacillus thuringiensis*-derived Cry5B has potent anthelmintic activity against *Ascaris suum*. PLoS Negl. Trop. Dis..

[30] Hu Y., Miller M., Zhang B., Nguyen T.T., Nielsen M.K., Aroian R.V. (2018). In vivo and in vitro studies of Cry5B and nicotinic acetylcholine receptor agonist anthelmintics reveal a powerful and unique combination therapy against intestinal nematode parasites. PLoS Negl. Trop. Dis..

[31] Lyons E.T., Drudge J.H., Tolliver S.C. (1990). Prevalence of some internal parasites found (1971-1989) in horses born on a farm in Central Kentucky. J. Equine Vet. Sci..

[32] Hu Y., Zhan B., Keegan B., Yiu Y.Y., Miller M.M., Jones K., Aroian R.V. (2012). Mechanistic and Single-Dose In Vivo Therapeutic Studies of Cry5B Anthelmintic Action against Hookworms. PLoS Negl. Trop. Dis..

[33] Hu Y., Xiao S.H., Aroian R.V. (2009). The new anthelmintic tribendimidine is an L-type (Levamisole and Pyrantel) nicotinic acetylcholine receptor agonist. PLoS Negl. Trop. Dis..

[34] Marcellino C., Gut J., Lim K.C., Singh R., McKerrow J., Sakanari J. (2012). WormAssay: A novel computer application for whole-plate motion-based screening of macroscopic parasites. PLoS Negl. Trop. Dis..

[35] PubChem. https://pubchem.ncbi.nlm.nih.gov/.

